# Supplementary figures and images for: Regression shrinkage and selection via least quantile shrinkage and selection operator
Source: PLoS One. 2023 Feb 16;18(2):e0266267. doi: 10.1371/journal.pone.0266267 (PMC9934385; doi:10.1371/journal.pone.0266267)

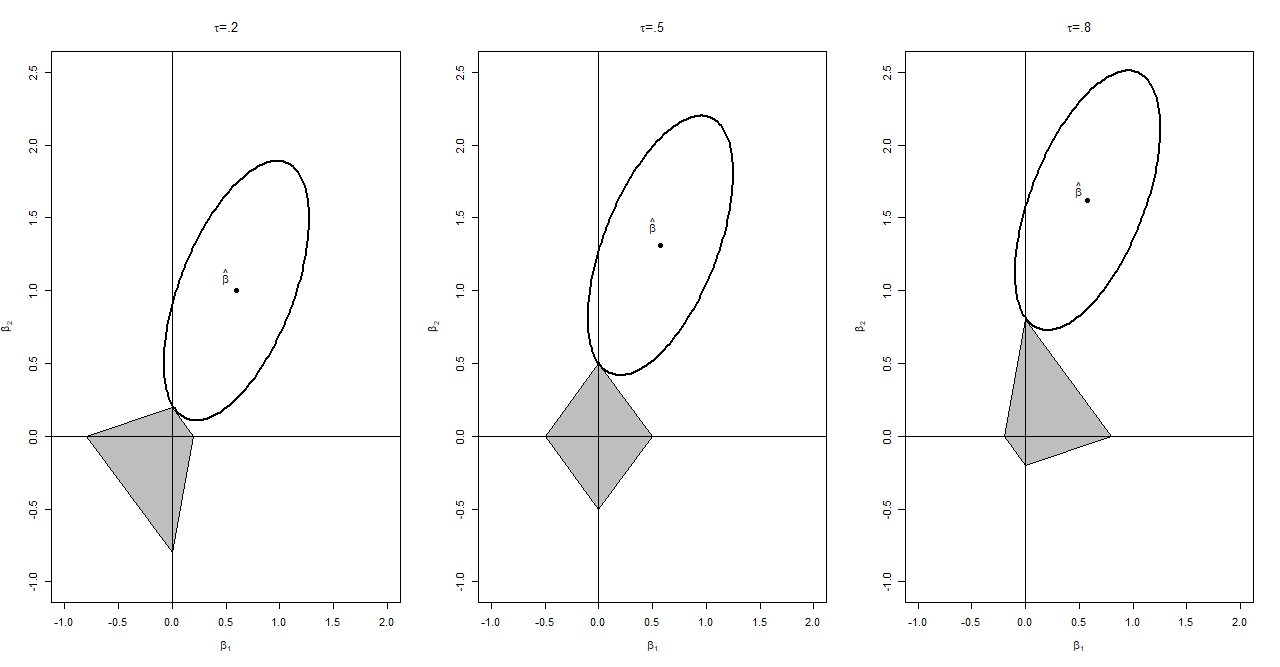

Supplement: S1 Fig — Plot of the lqsso estimators with τ = .2, τ = .5 (approximately equal to the lasso) and τ = .8 sketched at the left, middle and right panels, respectively, centered at OLS estimates. See the text for more details. (TIFF) [file pone.0266267.s001.tiff]

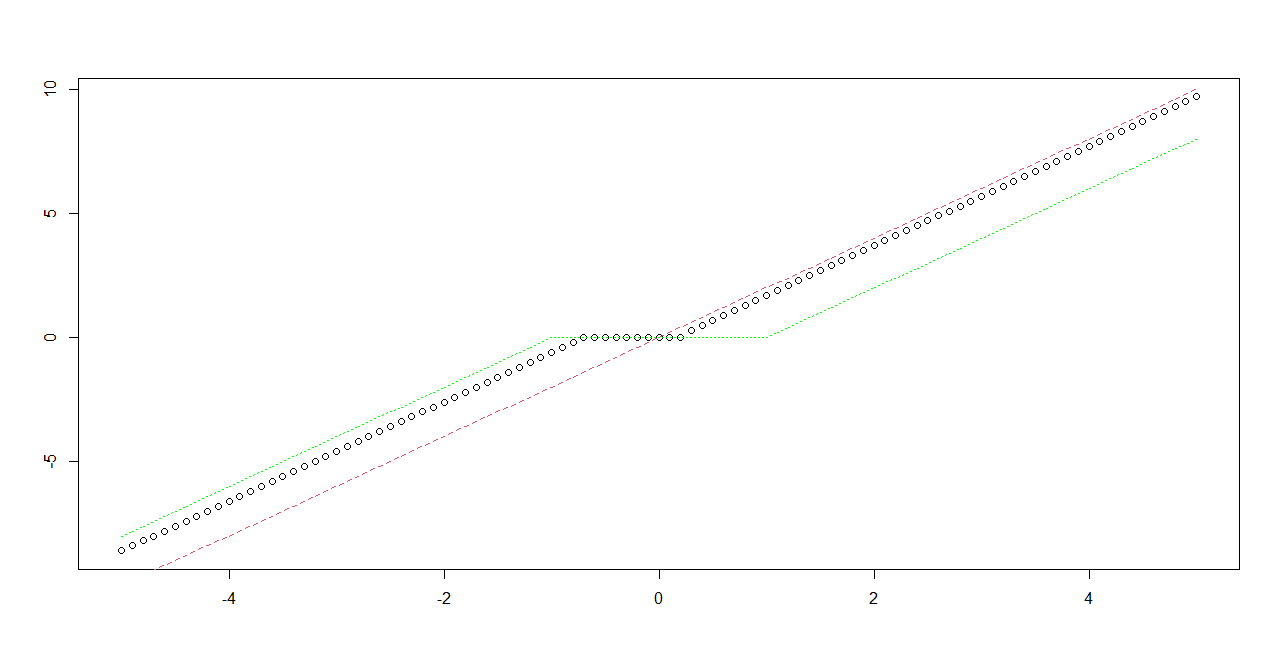

Supplement: S2 Fig — Plot of the lqsso penalty in comparison with the lasso and adaptive lasso penalty according to the plot C in Zou [6]. (TIFF) [file pone.0266267.s002.tiff]

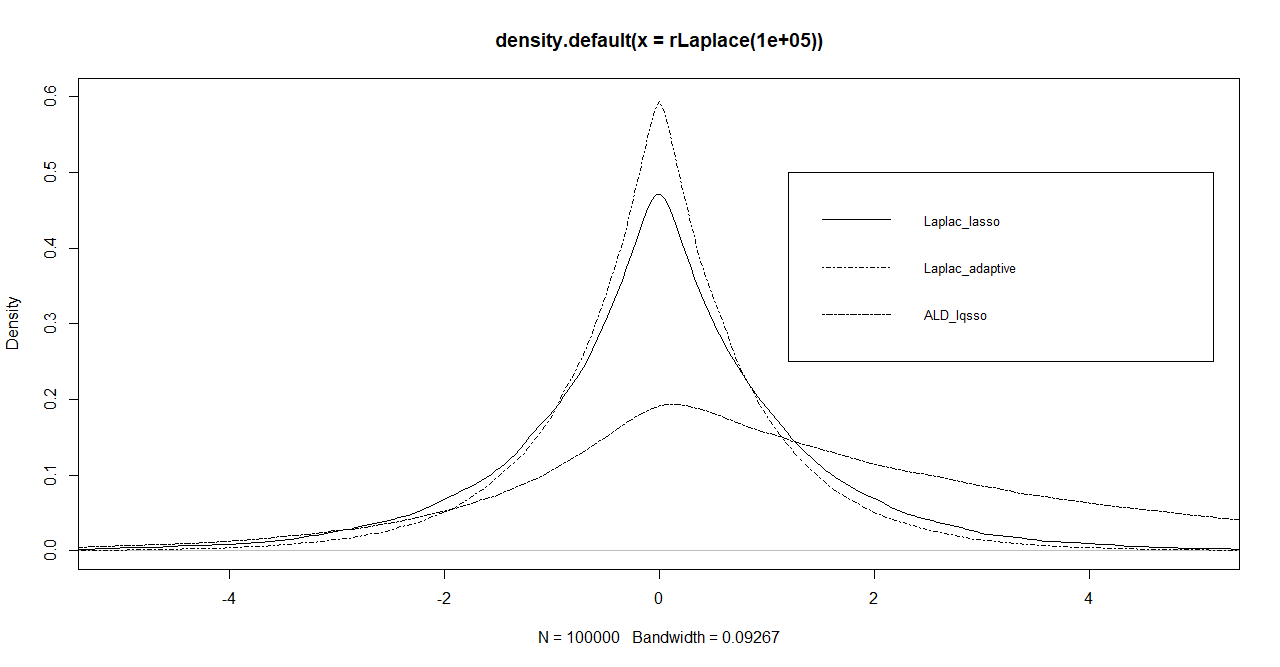

Supplement: S3 Fig — Plot of the lqsso penalty in comparison with the lasso and adaptive lasso penalty based on Bayesian approach. (TIFF) [file pone.0266267.s003.tiff]

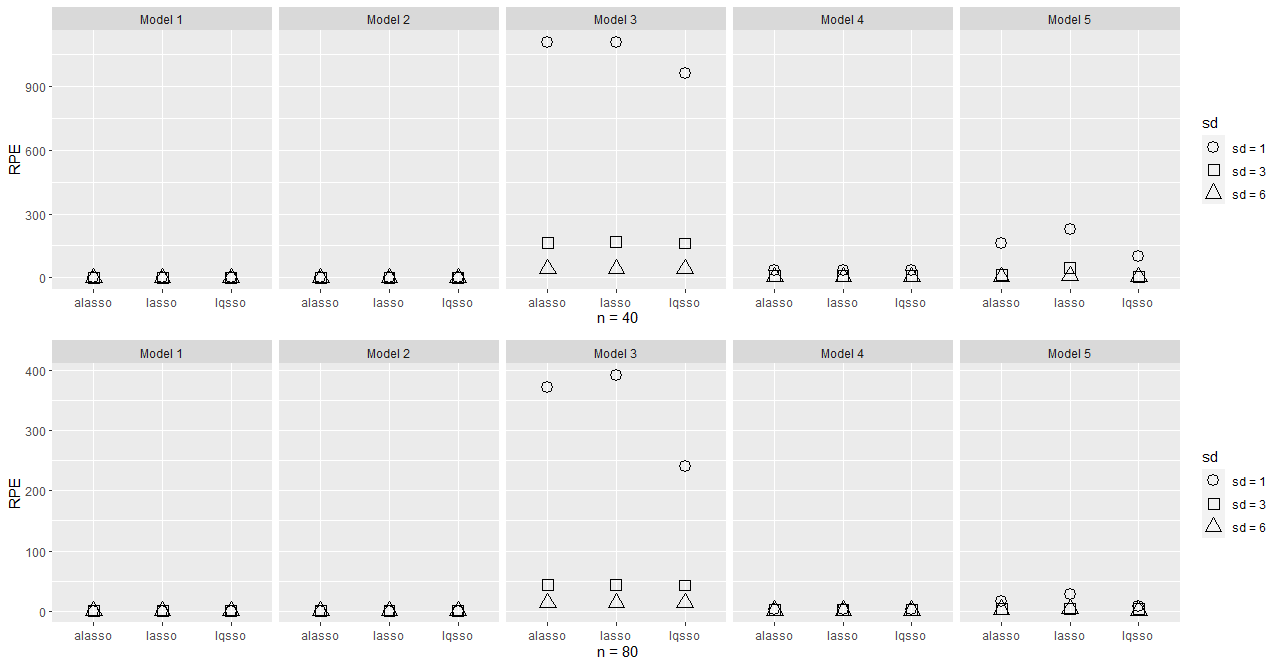

Supplement: S4 Fig — Compare methods in terms of the RPE criterion using different models, sample sizes (n) and the standard deviation (sd) of the error term. (PNG) [file pone.0266267.s004.png]

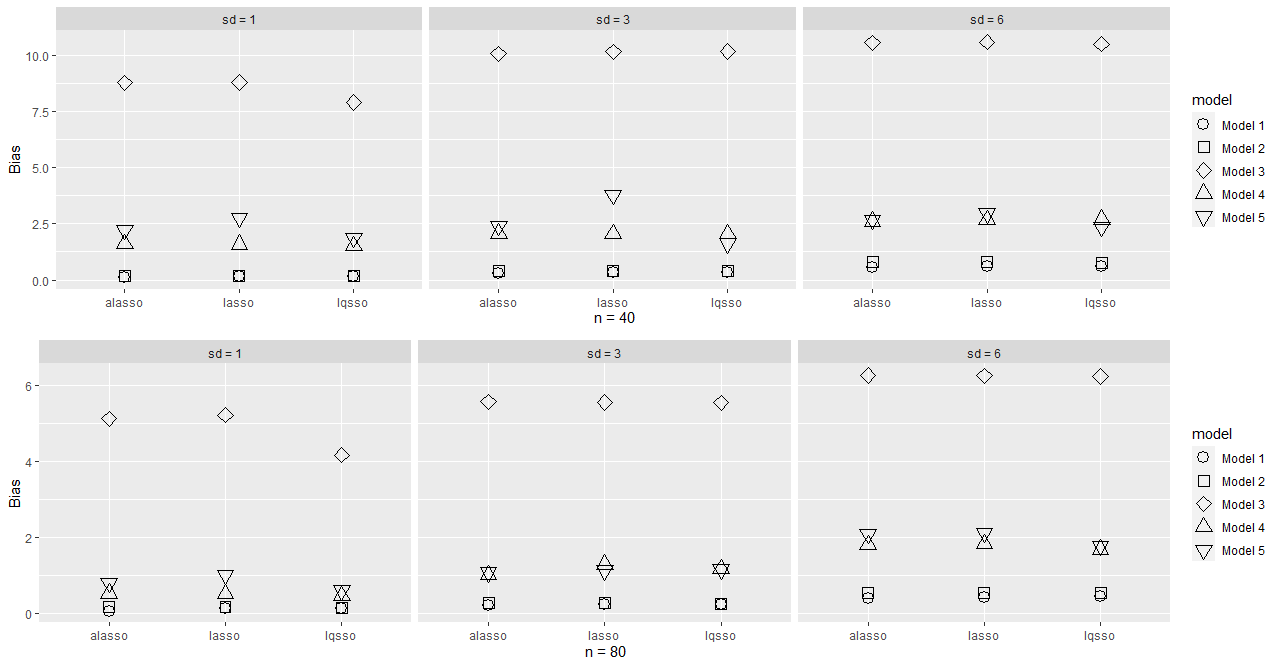

Supplement: S5 Fig — To compare methods in terms of the bias criterion using different models, sample sizes (n) and the standard deviation (sd) of the error term. (TIFF) [file pone.0266267.s005.tiff]

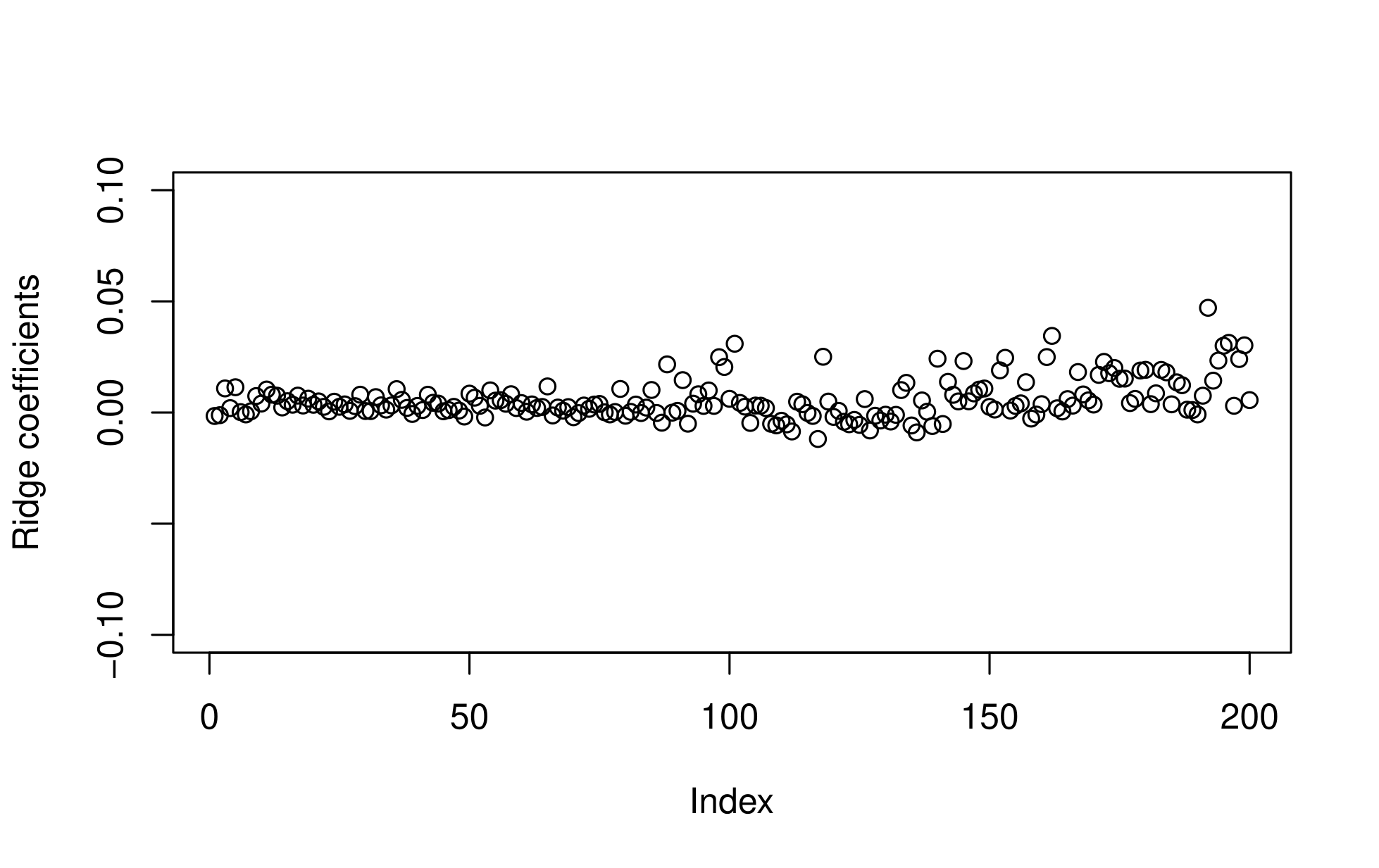

Supplement: S6 Fig — The plot shows the ridge estimates for the coefficients of the corresponding ridge regression model. (TIFF) [file pone.0266267.s006.tiff]
