# Supplementary material for: Regression shrinkage and selection via least quantile shrinkage and selection operator
Source: PLoS One. 2023 Feb 16;18(2):e0266267. doi: 10.1371/journal.pone.0266267 (PMC9934385; doi:10.1371/journal.pone.0266267)
Supplement: Attachment — Submitted filename: Response to Reviewers.docx [file pone.0266267.s007.docx]

Dear Professor Song

Academic Editor of PLOS ONE

The authors appreciate you and the reviewers for their valuable comments. Indeed, all hints help us to improve the quality of our manuscript. Below, we answered the reviewers’ comments and applied them appropriately to the revised version of our manuscript. Following your recommendation, we also provided a revised manuscript with the changes highlighted in yellow colors.

**Responses to Reviewer #1**

Major comments:

1) In the simulation studies section, the authors compared RPEs in Table 1 and 4, bias in Table 2 and 4, number of (in)correct selected variables in Table 5. However, the authors only provided the standard errors for RPEs in Table 1 and 4. I am wondering whether the authors could also provide standard errors for bias and number of (in)correct selected variables in other tables.

The standard errors have been reported for the biases and the number of (in)correct selected variables and they are added in the relevant tables.

2) For number of (in)correct selected variables in Table 5, I would recommend using the mean and standard errors instead of median, just to be consistent with the previous 4 tables. Are there any reasons to use median here?

Following Zou (2006), because the true numbers are inherently integer values, we prefer to report the median for the number of (in)correct selected variables. Moreover, as suggested, the standard errors of those numbers were also reported in the related tables.

3) Similarly, in Table 6, please provide the standard error for the cross validation so that audience can know the variability of the metric

The standard errors for the cross-validations were also reported in Table 6. We appreciate your suggesting this point.

Minor comments:

1) The 3rd paragraph in the Introduction section (From "One of the existing" to "for the coefficients") talks about the potential Bayesian understanding for the new approach. I would recommend moving it to the Discussion section or cut it shorter as it is beyond the function of the "Introduction" section.

Our Bayesian view of proposed idea became shorter in the Introduction, and more comments are added in the Conclusion section.

2) I would strongly recommend the authors to make the code of new method publicly available so that people from a broader community can utilize.

Thanks for this recommendation. However, we currently cannot share our code publicly because we’re still working on it for other research studies. Notably, we are improving the idea of quantile regression and mixed quantile regression with lqsso penalty.

3) The figures need to be improved. For example, in Fig 1, the scale has been distorted. In Fig 2, please get rid of the "_" in the figure labels.

The scales set in figure 1. Also, we corrected the order of \tau=0.2, 0.5 and 0.8 in this figure. Also, we removed the “_” sign in the Y label of the current version of figure 2.

4) In Table 1 to Table 6, I think the authors are providing the mean values. Please specify that instead of just putting "values" and let the readers turn back and forth to find out what the values mean.

Those were good points. Now, we replaced the “values” by the “mean values” in Table 1 to Table 6 except in Table 5, which is, in fact, the median.

**Responses to Reviewer #2**

1) When \tau=0.5, LQSSO is equivalent to LASSO. Since LASSO doesn't have oracle property in general, how could the oracle property of LQSSO be established? Is there some non-trivial necessary condition missing?

That is true that the Lqsso with \tau=0.5 is equivalent to Lasso graphically but this is not in reality. The reason is that the lqsso is approximately equal to the lasso. Specifically, the main difference between the two is that lqsso consists of the indicator function of OLS coefficients in the penalty component, making it different from LASSO if \tau=0.5.

2) Why is the proposed weight superior? Or break down to (1), Why does considering the sign of initial coefficients help? e.g., Given \tau=0.2, w=0.8 when the initial coefficient (usually OLS or Ridge estimates) is negative and w=0.2 when it is positive. (2) If the goal is to constrain the weight between 0 and 1, how about sth like w_j = 1 / (1 + exp(|\beta|))?

Compared with your proposed penalty w_j = 1 / (1 + exp(|\beta|)), lqsso relies on check function (\rho _{\tau}(.)). So it has closed-form and many representations to be used in various circumstances such as implementing an algorithm, invoking in a geometrical view, recalling a Bayesian view, using for proof of Oracle property, and calculating KKT conditions (closed form) in the quantile regression with lqsso penalty. Other representations are as follows.

The signs show the direction of the polygon in the figure and OLS coefficients in the penalty part. More precisely, OLS coefficients are based on a density function f(y|X), so it considers the mass not only at the middle of the density but also at their two tails, especially when f(.) is asymmetric. Hence it is more flexible more than lasso and adaptive.

Plot C in Zou (2006) is as follows for lqsso (black points). Note that Zou (2006) indicated them by a line in his graph, but we preferred to plot them as points for better illustration purposes. Ali, it is not clear and lasso (Green line). It shows according to the \tau value and its sign, lqsso line how much is closer to the red line and also on the positive or negative side.

Moreover, one can use the ALD as a prior distribution for the initial coefficients and obtain the estimations of the lqsso via computing the MAP estimator. This is the case if one considers the ALD as the prior distributions for the initial coefficients. Johnstone and Titterington (2009) also presented a Bayesian view to the lasso model by setting up the prior density for the coefficients as the Standard Laplace distribution. To do so, the zero coefficients will have a high probability compared to the non-zeros, and this feature would guarantee the sparsity. The plot given below shows this exciting property. To the best of our knowledge, the adaptive lasso has not been treated from a Bayesian perspective. We expect this gap can be filled via considering a re-scaled Standard Laplace distribution. This topic will be tackled in our future research.

Let us go back to your question, i.e., the effect of the signs. As mentioned, we should initially fix \tau, e.g., via invoking the cross-validation technique. Now, assume that \tau=0.8, where \beta_1>0 and less than 1 (consequently w_1>1). Then, we could assign a probability for this true coefficient and a reasonable probability for zero coefficients, i.e., sparsity. This idea owns to the signs.

Moreover, our weights will still be between 0 and 1. Note that it advocates the highest probability for zero for the adaptive case, as seen in the plot.

As a summary, the table below indicates where our proposed method shows superiority compared with other methods.

| Property | Lasso | Adaptive | Lqsso |
| --- | --- | --- | --- |
| Oracle |  | Yes | Yes |
| Bayesian view and rationale | Yes |  | Yes |
| Use lasso algorithm | Yes | Yes | Yes |
| Weights are based on random variables & observations |  | Yes | Yes |
| Geometry aspect | Yes |  | Yes |

3) Please provide literature or numerical or theoretical support for the claim: If the absolute of the initial coefficient is less than 1, the weight will be large leading to increase in bias.

The rationale behind this claim includes the following sequential statements:

a) If the absolute of the initial coefficient is less than 1, the weight will be large.

b) In a regular regression (OLS), there is no penalty part, bias is low and variance is high. By using a penalty, we sacrifice bias (has increased) to reduce variance i.e., bias-variance tradeoff. Hence, with adding a penalty, bias increases.

c) According to Buhlmann and Van De Geer (2011), if |\beta_ j,initial| is large, the adaptive Lasso employs a minor penalty (i.e. little shrinkage) for the jth coefficient (b_j) which implies less bias.

d) The topic has not already been appropriately addressed in the literature. Albeit, Zou (2006), B{\"u}hlmann and Van De Geer (2011) and Fan et al. (2009) suggested some easy solutions such as taking the initials coefficients equal to zero if that particular circumstance occurs.

So, considering a&b or (c&d, with less weights) support our claim.

4) Page 6: ''This is in contrast with other penalty proposed so far in which the response's quantile did not play a role in the penalty.'' So, how does LQSSO include Y's quantile in the penalty with w_j = (1-\tau)*I(\beta_j <= 0) + \tau*I(\beta_j > 0)?

We should point out that our proposed idea is not directly based on response's quantile. But, generally, the regression modeling set-up is related to the quantity E(y|X,\beta,\tau) which takes the randomness of Y into account. Moreover, we do see that the penalty term utilized the estimate of the OLS coefficients and these latter values are derived via the density function f(y|X), or directly speaking the randomness of Y. Hence, our considered check function, i.e. \rho, inherits Y’s quantile.

5) Fig 1 is a nice illustration, while (1) Given one \tau, suppose it is the \tau selected after tuning, the constraint region is still a trapezoid, why does it provide more comprehensive insight than LASSO? (2) Please also plot a figure similar to Fig 1(c) of Zou (2006). I guess it is a horizontal shift of Fig 1(c). So this is related to Question 2, what does LQSSO gain or lose from the shift?

The answer to this comment is provided in replying to the comment 2.

6) Please check statements and language, some are confusing. e.g., Page 7, last paragraph: ''when the initial weights for the coefficients in the adaptive LASSO are less than one'', what does it refer to, w_j or \beta_j?

The correct sentence is “when the initial coefficients for the weights in the adaptive lasso are less than one.” We corrected this in the revised version.

The best regards

Mousa
