# Supplementary material for: Regression shrinkage and selection via least quantile shrinkage and selection operator
Source: PLoS One. 2023 Feb 16;18(2):e0266267. doi: 10.1371/journal.pone.0266267 (PMC9934385; doi:10.1371/journal.pone.0266267)
Supplement: Attachment — Submitted filename: Response to Reviewers.docx [file pone.0266267.s008.docx]

Dear Professor Song

Academic Editor of PLOS ONE

The authors appreciate you and the reviewers for all valuable comments and suggestions raised in the second round of reviewing our article. Indeed, all comments were beneficial and help us to improve the quality of our manuscript. We revised the way of English writing in this new version and attempted to provide well organized, clear and understandable sentences. Also, we tried to address the important points raised by the Reviewer 2. Moreover, following the journal’s policy, we also provided a revised manuscript with the changes highlighted in yellow colors. We hope this second revised version of the manuscript fits with what have been asked and suggested.

As we understood, the Reviewer 1is fully satisfied with the changes made in our first revised paper as well as the responses to her/his comments. The Reviewer 2, but, was mostly concerned about the questions (we assume those were default of the PlOS-One) 2 and 6 in your previous email. She (he) was partly happy with the question 2 “2. Is the manuscript technically sound, and do the data support the conclusions?”. Hence, we attempted to improve the paper to fit with this request. Considering the question 6 “Review Comments to the Author “, we tried to fit ourselves with her/his request in the points (1) and (2) and set all necessary materials in the paper, particularly where the concepts should have been cleared. We hope the changes made in those relevant pages, sections and expressions are well enough to convince her/him.

Sincerely Yours

Mousa
